# Prognostic ability of the German version of the STarT Back tool: analysis of 12-month follow-up data from a randomized controlled trial

**DOI:** 10.1186/s12891-019-2467-6

**Published:** 2019-02-28

**Authors:** Sven Karstens, Katja Krug, Heiner Raspe, Max Wunderlich, Martin Hochheim, Stefanie Joos, Angelika Hüppe

**Affiliations:** 1 Department of Computer Science, Therapeutic Sciences, Trier University of applied Science, Trier, Germany; 2 Department of General Practice, University of Tuebingen, Tuebingen, Germany; 3 Department of General Practice and Health Services Research, University Hospital Heidelberg, Heidelberg, Germany; 4 Institute of Social Medicine and Epidemiology, University of Luebeck, Luebeck, Germany; 5 Central Krankenversicherung AG, Cologne, Germany; 6 Generali Health Solutions GmbH, Cologne, Germany

**Keywords:** Back pain, Prognosis, STarT Back tool, Psychometrics, Primary health care, Questionnaire

## Abstract

**Background:**

Stratified care is an up-to-date treatment approach suggested for patients with back pain in several guidelines. A comprehensively studied stratification instrument is the STarT Back Tool (SBT). It was developed to stratify patients with back pain into three subgroups, according to their risk of persistent disabling symptoms. The primary aim was to analyse the disability differences in patients with back pain 12 months after inclusion according to the subgroups determined at baseline using the German version of the SBT (STarT-G). Moreover, the potential to improve prognosis for disability by adding further predictor variables, an analysis for differences in pain intensity according to the STarT-Classification, and discriminative ability were investigated.

**Methods:**

Data from the control group of a randomized controlled trial were analysed. Trial participants were members of a private medical insurance with a minimum age of 18 and indicated as having persistent back pain. Measurements were made for the risk of back pain chronification using the STarT-G, disability (as primary outcome) and back pain intensity with the Chronic Pain Grade Scale (CPGS), health-related quality of life with the SF-12, psychological distress with the Patient Health Questionnaire-4 (PHQ-4) and physical activity. Analysis of variance (ANOVA), multiple linear regression, and area under the curve (AUC) analysis were conducted.

**Results:**

The mean age of the 294 participants was 53.5 (SD 8.7) years, and 38% were female. The ANOVA for disability and pain showed significant differences (*p* < 0.01) among the risk groups at 12 months. Post hoc Tukey tests revealed significant differences among all three risk groups for every comparison for both outcomes. AUC for STarT-G’s ability to discriminate reference standard ‘cases’ for chronic pain status at 12 months was 0.79. A prognostic model including the STarT-Classification, the variables global health, and disability at baseline explained 45% of the variance in disability at 12 months.

**Conclusions:**

Disability differences in patients with back pain after a period of 12 months are in accordance with the subgroups determined using the STarT-G at baseline. Results should be confirmed in a study developed with the primary aim to investigate those differences.

## Background

Back pain is one of the most prevalent symptoms encountered in primary care [1]. Its treatment is challenging for primary care practitioners, such as physiotherapists or general practitioners [2, 3]. It has been discussed that determining the prognosis of back pain is a priority for research and practice and may have the potential to supersede diagnosis in its relevance [4]. For the purpose of prediction, several risk factors were identified and differentiated as being modifiable (e.g. psychological factors) or non-modifiable (e.g. pain history) [5–7]. Despite this knowledge, and although planning of treatment on such a basis seems ineffective, prediction in clinical practice mainly relies on experience and clinical judgement [1, 8].

Several prognosis-based approaches to subgroup patients with back pain have been developed [9]. One which has demonstrated feasibility, clinical applicability and cost-effectiveness is the STarT Back Approach (STarT = Subgroups for Targeted Treatment) [10, 11]. To establish the prognosis of an unfavourable treatment outcome for patients with back pain, biomedical and modifiable psychosocial factors are determined using the STarT Back Tool (SBT) [6]. With nine items and mainly dichotomous answer categories, it is easy to complete [6] (http://www.keele.ac.uk/sbst/startbacktool/downloads/), and its utilisation is recommended by national and international guidelines [12–14]. Evaluation of the tool results in a biopsychosocial total score and a sub-score focusing on psychosocial constructs. A classification comprised of low-, medium- and high-risk subgroups can be derived. For groups at higher risk more severe disability is expected over time [6].

The SBT was originally developed in the UK [6]. Following an internationally agreed upon process, it was cross-culturally adapted for German-speaking countries, and several important psychometric properties were examined [15–17]. The German version is called STarT-G [17]. Although the properties are not in total agreement with those of the original version, they can be considered acceptable to good [16, 17]. Predictive ability has been investigated internationally in a few studies, but not for the German version [18–20].

The SBT was specifically developed for primary care and supports physicians and therapists in the clinical decision-making process. In addition to the advantage of better quality of care, in the sense of a more patient-oriented approach, it entails possibilities for cost savings, and, as a result of the utilisation of the tool, a reduction of unnecessary treatment may lead to a reduced burden on the patient [10, 11, 21]. In addition to its main area of application in clinical practice, the tool could be used by stakeholders such as health insurances to provide clients with better targeted suggestions for prevention. A corresponding programme called ‘initiative.rücken’ (‘back.initiative’) was developed by a German private health insurance company. It includes treatment by an interdisciplinary network of therapists, and individual coaching by phone is offered. An evaluation study was planned in parallel with the implementation of this proactive integrated treatment programme, and among other instruments, the STarT-G was applied. However, since the predictive ability of the German version has yet to be established, the STarT-Classification was only used for the purpose of description and not to derive treatment recommendations [22].

The primary aim of the a priori planned analysis of data from a pragmatic trial in health services research was to analyse the disability differences in patients with back pain 12 months after inclusion, according to the subgroups determined at baseline using the STarT-G. Moreover, several secondary aims were set out. The potential for improving prognosis for disability by adding further psychosocial and lifestyle variables was investigated. It was analysed whether the patients’ pain intensity 12 months after inclusion differs in accordance with the subgroups determined at baseline, and the properties for discriminative ability and floor and ceiling effects were determined.

## Methods

Baseline and 12-month follow-up data of a Zelen randomized controlled trial (RCT) conducted in 2015 to evaluate the efficacy of a health programme for persistent back pain were analysed. Participants of the RCT were members of a German private medical insurance with a minimum age of 18 and with back pain persisting for a period of more than 3 months. For case identification, at least two entries, according to ICD-10 codes M40–M54, had to be given in the insurance database. Additionally, one case of temporary work disability in the previous 12 months was due to ICD codes M40–M54, two opioid prescriptions or data for other specified diagnoses. Inclusion and exclusion criteria can be found in Table 1 (for an extended version with category names, see Appendix 1). A random sample of eligible insured persons was randomly allocated to the trial groups prior to their giving consent. The analysed data included in this study came from those allocated to the control group, and those people were invited to participate in a survey dealing with persistent back pain while receiving usual care. In this context, ‘usual care’ indicates that patients received care at the behest of their treating general practitioner or specialist, and the investigators had no influence on the treatment. All persons who gave written informed consent received online questionnaires at baseline and again at 12 months after inclusion. Ethical approval for the trial was granted by the Ethics Committee of the University of Luebeck (registration ID: 14/249). Analysing the properties of the STarT-G was planned at the same time as the development of the primary RCT, which is described in more detail in Hüppe et al. [13].

**Table 1:** In- and exclusion criteria checked in the insurance database

Inclusion	- Age ≥ 18 years - ≥ 2 cases of settlement data due to ICD codes M40-M54 - Additionally, one of the following three - ≥ 1 cases of temporary work disability in the past 12 months due to ICD codes M40-M54, - two opioid prescriptions or - settlement data due to one of the following ICD-10 codes: F32.-, F33.-, F34.1–34.9, F38.-, F41.2, F43.2, F45.4, F48.0, F54, F62.8
Exclusion	- Care level II or III (long-term care insurance act, SGBXI) - ICD codes in the past 12 months (settlement data ≥2 for the same diagnosis or ≥ 5 different) - B16.-16.9, B17.1, B20–24, D00-D09.9, F00.-F09, F10.0–19.9 (not F17.0–9), F20.-F29, F30.-F30.9, F31.-F31.9, F42.-F42.9, F60.-F60.9, G00–09, G10, G13.-G13.8, G23.-G23.9, G30.-32.8, G36.-G36.9, G37.9, G92, G93.-G93.9, H54.0, H91.3, I64, K74.-K74.6, N18.0, N18.3–4, N18.9, R54

The investigated instrument is the STarT-G. It consists of nine items and is used to determine an individual’s prognosis related to disability. The first four items relate to biomedical factors and the remaining five identify modifiable psychosocial risk factors [6, 19]. A total-score ranging from 0 to 9 points and a sub-score for the psychosocial risk factors ranging from 0 to 5 points were calculated. Patients were then allocated to one of three prognostic groups using established scoring cut-offs (low-risk: total score ≤ 3 points; medium-risk: total score > 3 and sub-score < 4 points; high-risk: sub-score ≥ 4 points). Disability and back pain intensity were measured using the Chronic Pain Grade Scale (CPGS). The CPGS disability score served as the patient’s primary outcome (CPGS-DS). It shows back-pain-related disability, determined by the amount the pain interfered with daily, social and work activities and ranges from 0 to 100, with higher scores indicating more disability [23, 24]. Additionally, health-related quality of life, psychological distress and physical activity were measured. For this purpose, the Short-Form-12 Physical and Mental Health Summary Scales (SF-12; range 0 to 100, higher score indicating better health) [25], the Patient Health Questionnaire-4 (PHQ-4; 0 to 12 points, higher score indicating more severe psychological distress) [26] and activity-specific items according to DEGS1 were used (dichotomized into rather inactive = 0, rather active = 1; for details see [27]).

### Statistics

Descriptive statistics were calculated to characterize the study population, and completeness of follow-up data was stated. To address the primary objective of the analysis, an ANOVA using the STarT-Classification as a grouping variable and the CPGS-DS at 12 months as the outcome variable was conducted [19, 23]. Tukey tests were done post hoc to analyse the mean differences among the three subgroups. Deviation measures (SD) were reported to estimate the variability of disability within the risk groups at 12 months. This procedure, without the inclusion of covariates such as age or other scores, was chosen as the primary approach because the 9-item STarT-G is simple, allowing for use in clinical practice as a single predictive instrument.

While the SBT comprises only modifiable risk factors, the literature states that inclusion of other factors could improve prediction [20, 28]. Therefore, the potential to improve prognoses for disability by adding further variables was investigated as a secondary approach. This was only done for our primary outcome disability at 12 months based on the CPGS-DS as the outcome variable. The procedure encompassed two main steps. First, univariate linear regression analyses were carried out to check for, at least, a minimal dependence. Considered were disability at baseline, depression/anxiety based on the PHQ-4, patient self-prognosis for workability, physical activity and global health. All these variables were derived from the survey. STarT-Classification was included as two dummy coded variables, medium and high risk (patients with a medium and high risk coded as 1 respectively), in each case against the other two groups (coded as 0). Patients with a low-risk were used as the reference, being 0 in both dummy variables. For the variables to pass to step two (multiple linear regression analysis), a result of *p* ≤ 0.2 was necessary from univariate analysis. For the multiple linear regression analysis, the variables were included block-wise. First, the STarT-Classification variables were included to determine the variance explained by the STarT-G. Second, the covariates from the univariate analyses were included before the final model was determined by applying a backward stepwise method.

For pain intensity at 12 months as the outcome variable, an ANOVA was carried out as described for the primary objective.

To quantify discriminative ability for patients with different disability and depression/anxiety statuses based on the PHQ-4, receiver operating characteristic (ROC) curves with area under the curve (AUC) and 95% confidence interval (CI) for CPGS against STarT-G total score and PHQ-4 against STarT-G sub-score were computed for baseline and at 12 months. The CPGS was calculated following the standards given by von Korff et al. [23]. To compute ROC curves, a dichotomous reference standard is needed. To define reference standard ‘cases’, the GCPS-categories established by von Korff et al. [23] were used: Grades 0, I and II (low disability) versus Grades III and IV (high disability cases). For definition of PHQ-4 cases, the predefined groups of none and mild versus moderate and strong burden were combined. Adjectives that can be used to describe AUC values have been proposed by Hosmer and Lemeshow with an AUC = 0.5 indicating ‘no discrimination’, 0.7 to < 0.8 as ‘acceptable discrimination’, 0.8 to 0.9 as ‘excellent discrimination’ and > 0.9 as ‘outstanding discrimination’ [29].

Additional information on the relationships among the instruments was acquired by calculating Spearman correlation coefficients for the STarT-G total and sub-scores against the CGPS-DS, the PHQ-4-score and the SF-12 sub-scores for physical function and mental health, based on the original metrically scaled variables. As stated for the original version, higher correlations were expected for the total score versus the physical aspects and for the sub-score versus the psychological aspects [6].

Floor and ceiling effects were defined as present if more than 15% of the responders achieved the lowest or highest possible STarT-G total score [30].

Statistical tests were two-sided, and a significance level of alpha = 5% was used, unless otherwise stated. The analyses were performed using IBM SPSS Statistics 25.0 (IBM, Armonk, NY, USA). Figures were produced using the R language and environment for statistical computing software version 3.4.1 [31].

## Results

From the database of the private medical insurance company, a random sample of 1499 eligible members was randomly allocated to the control group and invited to take part in a survey. Of those, 294 gave informed consent and answered the baseline questionnaire, and 243 (82.7%) participated in the 12-month follow-up. The mean age of participants was 53.5 (SD 8.7) years, and 38% were female. There were no significant differences for age (*p* = 0.56) and gender (*p* = 0.12) between responders and non-responders. The mean STarT-G total and sub-scores were 3.2 (SD 2.3) and 1.3 (SD 1.4), respectively. The risk group distribution was 62.6% for low, 27.6% for medium and 9.9% for high risk. Further baseline values are given in Table 2.

**Table 2:** Characteristics of the study population

Mean age in years (SD) n	53.5 (8.65)	293
Gender female %, n	38.1	112
Total		294
Mean Body-Mass-Index in kg/m^2^ (SD) n	27.2 (4.91)	293
Education %, n		
up to 9 years	12.8	37
10 to 11 years	41.3	119
12 to 13 years	45.8	132
Total		288
Physically active per week %, n		
Never	14.6	43
1 day	16.7	49
≥ 2 days	68.7	202
Total		294
Mean (SD) pain intensity (0–100) n	44.7 (21.24)	294
Mean (SD) CPGS-DS (0–100) n	36.8 (24.63)	294
CPGS %, n		
chronic pain grade low disability (Stadium 0 - II)	58.5	172
chronic pain grade high disability (Stadium III + IV)	41.5	122
Total		294
SF-12 Physical Health, mean (SD) n	40.3 (11.45)	294
SF-12 Mental Health, mean (SD) n	44.5 (12.06)	294
General health n, %		
less good/poor	38.1	112
good/excellent	61.9	182
Total		294
PHQ-4, mean (SD) n	3.3(2.75)	294
Working until pension (patient self-prognosis) %, n		
sure/probably yes	56.02	149
unsure/probably no/certainly not	43.98	117
Total		266
Mean STarT-G total (SD) n	3.2 (2.29)	294
Mean STarT-G subscale (SD) n	1.3 (1.42)	294
STarT risk group %, n		
Low risk	62.6	184
Medium risk	27.6	81
High risk	9.9	29
Total		294

*CPGS* Chronic Pain Grade Scale, *DS* disability score, *SF-12* Short Form-12 Health Survey, *STarT* Subgroups for Targeted Treatment Back Tool, *STarT-G* German version of the STarT-Back Tool, *PHQ-4* Patient Health Questionnaire (4 items). *n* = 294

**Fig. 1:**
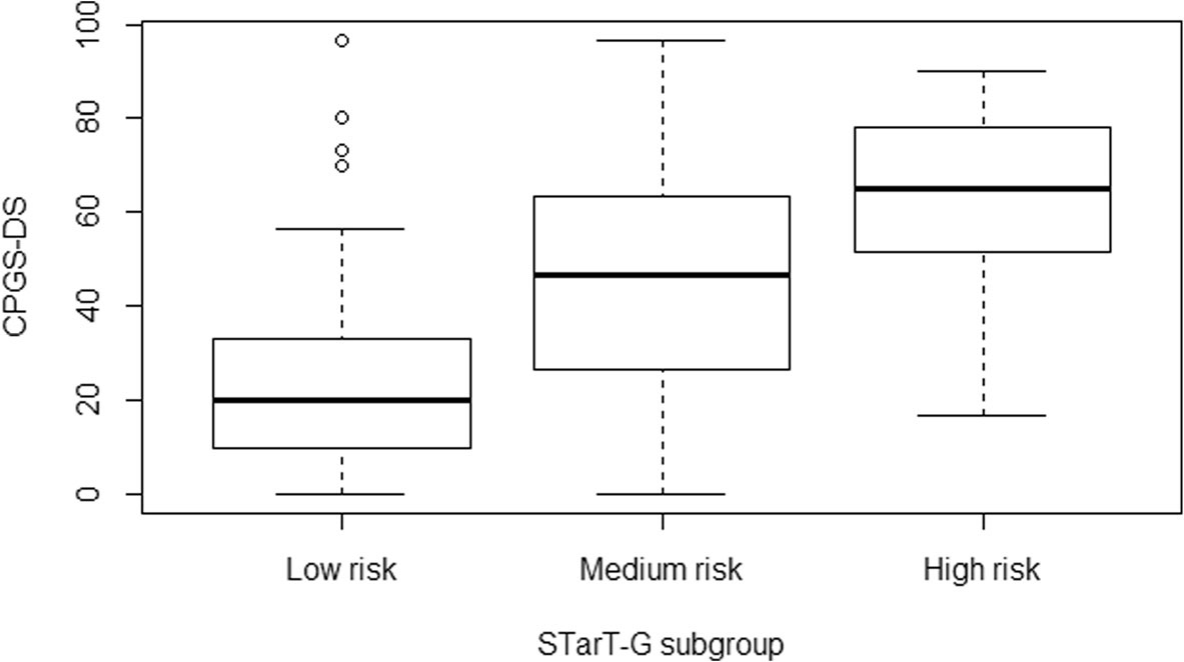
Boxplots for STarT-G subgroups low-, medium- and high-risk groups versus CPGS at 12 months. CPGS = Chronic Pain Grade Scale, DS = disability score, STarT-G = German version of the STarT-Back Tool. *n* = 243

The ANOVA for disability at 12 months indicated significant differences (df_total_ = 242, F = 51.7, *p* < 0.001) among the groups (Fig. 1). Post hoc Tukey tests revealed significant differences among all three risk groups for every comparison (Table 3).

**Table 3:** Post hoc Tukey analyses disability and pain intensity at 12 months

	Mean (SD)	Mean Difference	*p*
CPGS-DS at 12 months/ STarT group			
Low-Risk	24.10 (20.06)	21.86	< 0.001
Medium-Risk	45.96 (22.72)		
Medium-Risk	45.96 (22.72)	17.10	0.002
High-Risk	63.06 (19.83)		
Low-Risk	24.10 (20.06)	38.96	< 0.001
High-Risk	63.06 (19.83)		
Pain intensity at 12 months/ STarT group			
Low-Risk	35.21 (19.06)	19.74	< 0.001
Medium-Risk	54.95 (16.07)		
Medium-Risk	54.95 (16.07)	12.41	0.012
High-Risk	67.36 (16.59)		
Low-Risk	35.21 (19.06)	32.15	< 0.001
High-Risk	67.36 (16.59)		

*CPGS* Chronic Pain Grade Scale, *DS* disability score, *STarT* Subgroups for Targeted Treatment Back Tool

*n* = 243

For the multivariable regression analysis, four variables were adopted along with the STarT-Classification variables. Following the univariate step the *p*-values of the variables patient self-prognosis for workability, global health, disability at baseline and depression/anxiety, satisfied the set threshold of *p* ≤ 0.2 (Table 4). As a result of the first block of the regression analysis with only the STarT-Classification variables included, 28% of the variance in disability at 12 months was explained (adjusted R^2^). During the backward stepwise procedure, subjective work prognosis and depression/anxiety were excluded. The final model included the STarT-Classification variables, global health and disability at baseline (Table 5). ANOVA for the final regression model resulted in *p* < 0.001. The model predicted 45% of the variance in disability at 12 months (adjusted R^2^). The resulting model reads as follows: Disability at 12 months (CPGS-DS) = -5.61 + 5.59*STarT group medium risk + 14.26*STarT group high risk + 0.41*Disability at baseline + 6.44*General health. Semi-partial correlation coefficients ranged from 0.08 to 0.28.

**Table 4:** Predictors for disability (CPGS-DS) at 12 months: Results of univariate Regression Analysis

	Unstandardized Coefficients			
Prognostic factor	B	Beta	*p*	CI for B low, high
STarT group medium risk	16.58	0.30	< 0.001	9.85, 23.31
STarT group high risk	32.37	0.39	< 0.001	22.69, 42.05
CPGS-DS baseline	0.69	0.68	< 0.001	0.59, 0.78
PHQ-4	3.27	0.38	< 0.001	2.26, 4.29
Subjective prognosis work	7.77	0.40	< 0.001	5.38, 10.15
General health	17.29	0.55	< 0.001	13.99, 20.61
*Physical activity*	*1.00*	*0.70*	*0.25*	*−0.72, 2.72*

*CI* 95% confidence interval, *CPGS* Chronic Pain Grade Scale, *DS* disability score, *STarT* Subgroups for Targeted Treatment Back Tool, *PHQ-4* Patient Health Questionnaire (4 items), italicised indicating *p* > 0.2

**Table 5:** Predictors for disability (CPGS-DS) at 12 months: Results of the finale modle from multiple linear regression analysis

	Unstandardised Coefficients				
	B	Beta	*p*	CI for B low, high	Semi-part
(Constant)	−5.61		0.320	−16.68, 5.47	
Medium-risk STarT group	5.59	0.10	0.098	−1.05, 12.23	0.08
High-risk STarT group	14.26	0.17	0.004	4.50, 24.02	0.14
CPGS-DS baseline	0.41	0.40	< 0.001	0.26, 0.55	0.28
General health	6.44	0.21	0.001	2.51, 10.37	0.16

*CI* 95% confidence interval, *CPGS* Chronic Pain Grade Scale, *DS* disability score, *STarT* Subgroups for Targeted Treatment Back Tool, *Semi-part* semi-partial correlation

*n* = 219

The ANOVA for pain intensity at 12 months indicated significant differences between the risk groups (df_total_ = 242, F = 50.3, *p* < 0.001). Post hoc Tukey tests revealed significant differences for every comparison among all three risk groups (Table 3).

**Fig. 2:**
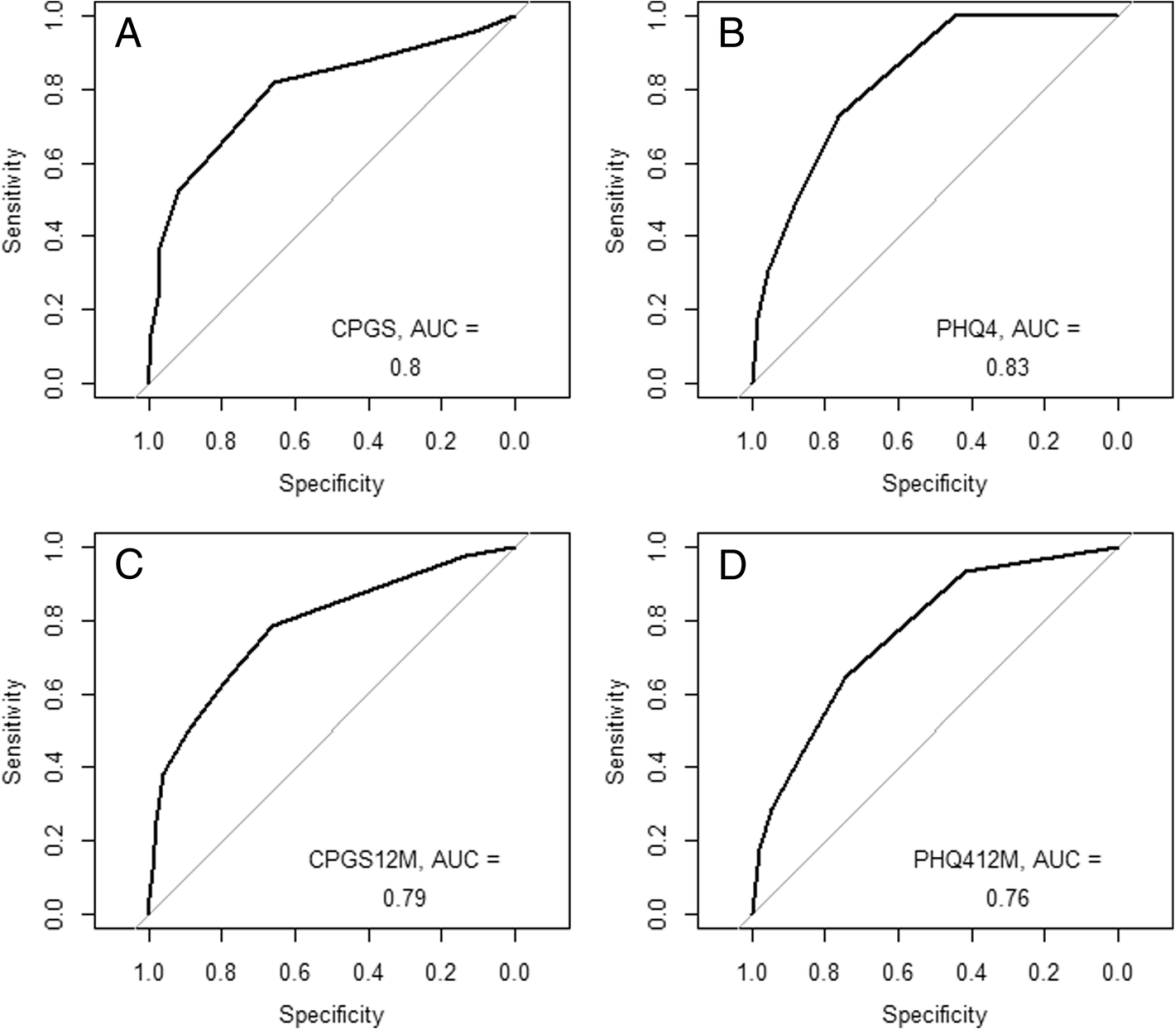
**a** to **d** Receiver operating characteristic curves. CPGS versus STarT-G total score and PHQ-4 versus STarT-G sub-score (baseline and 12 months). CPGS = Chronic Pain Grade Scale (dichotomized: Grade 0,I and II versus Grade III and IV), STarT-G = German version of the STarT-Back Tool, PHQ4 = Patient Health Questionnaire (4 items, dichotomized: none and mild versus moderate and severe psychological distress), 12 M = at 12 months. n_A_ = 294, n_B_ = 294, n_C_ = 242, n_D_ = 243

The AUC for STarT-G’s ability to discriminate reference standard ‘cases’ at baseline/12 months was 0.80 (95% CI 0.74, 0.85)/0.79 (95% CI 0.73, 0.85) for disability and 0.83 (95% CI 0.78, 0.88)/0.76 (95% CI 0.69, 0.84) for depression/anxiety, indicating acceptable to excellent discrimination (Fig. 2a to d).

Spearman coefficients for the STarT-G total and sub-score versus CPGS-DS, SF-12 Physical Health, SF-12 Mental Health and PHQ-4 scores are given in Table 6 (for graphical relationships between the STarT-G total score and CGPS-DS and between the STarT-G sub-score and PHQ-4, see Appendix 2).

**Table 6:** Correlation coefficients

		CPGS-DS	PHQ-4	SF-12 Physical Health	SF-12 Mental Health
START-G total score	rho	0.70	0.53	−0.65	−0.42
START-G sub-score	rho	0.61	0.58	−0.51	−0.51

*Rho* Spearman’s rho, *CPGS* Chronic Pain Grade Scale, *DS* disability score, *STarT-G* German version of the STarT-Back Tool, *PHQ-4* Patient Health Questionnaire (4 items)

*n* = 294, all correlations are significant; *p* < 0.001 in each case (two-tailed)

No floor or ceiling effects were found (8.2%, *n* = 24 patients with 0 points; 2.0%, *n =* 6 patients with 9 points).

## Discussion

In this study, data from nearly 300 patients were analysed. Used on its own, the STarT-Classification was of great value to predict differences in back-related disability 12 months after recruitment. By including additional variables in a prognostic model, it was possible to explain nearly half of the variance in disability at 12 months. Moreover, properties already determined in previous studies were confirmed, in part, with even higher coefficient values in the present sample [18, 19].

Primary users of the SBT are physiotherapists and primary care physicians [2, 3, 10]. In addition, utilisation of the tool by health insurance companies might have the potential for a more targeted disbursement of resources. Knowledge from previous research about the psychometric properties of the tool have been given, although not for its prognostic ability [18, 19]. Since such information is vital for clinicians [20], as well as for institutions, the given results have the potential to foster effective implementation. For health insurance companies, the presented analyses indicate possibilities for utilization of the instrument in their field.

Comparing the risk group distribution from the present study, which had many low-risk and a few high-risk patients, with those from other studies conducted in German-speaking countries, differences can be observed [6, 18, 19]. Various factors might influence the distribution. One might be the time point of administration [32–34]. Beneciuk et al. determined that more than three-quarters of high-risk patients changed categories after 4 weeks [33]. An alternative explanation for the differences in risk group distribution might be the recruitment strategy used for the study to provide the data analysed in this article. Participants were selected from the database of a health insurance and not in routine care.

The strength of the SBT is that the prognosis can be derived in clinical practice without gathering covariates. This approach worked well in this study’s chosen sample and is, therefore, in line with both the results from the developers’ external sample and another study including patients with chronic complaints [6, 21]. In contrast, Kongsted et al. used the SBT without covariates and described low accuracies for the Danish version of the tool [8]. In the present study, multiple linear regression analysis was only used as a secondary approach, but inclusion of covariates considerably improved prediction leading to an explanation of nearly half of the variance. Toh et al. stated that covariates were needed to obtain a better predictive model for pain scores at follow-up, whereas Medeiros et al. directly chose to include covariates, resulting in no predictive capability when using the baseline STarT-Classification [28, 35]. Consequently, the effect of including covariates needs further examination, while also remembering that the aim is to keep prediction as simple as possible for practice by focusing on modifiable factors.

A consideration of the results from the regression analysis revealed that other factors, as well as the STarT-classification, provided predictive information; here, these were disability at baseline and general health. The relevance of baseline scores is a common phenomenon in the literature [7, 32, 33] and is underlined by the determined semi-partial correlation coefficient in this study. In regard to general health, one simple item was identified which should be kept in mind for future predictive models. Also, the result indicating that the baseline score is a prominent predictor agrees with results from other studies [7, 32]. In the presented analysis, the STarT-Classification was used. Other researchers discuss the potential of the sub-score which might lead to different conclusions [28]. To improve predictive ability, different researchers have suggested a repeated application of the SBT, e.g. pre- and post-treatment, including the notion of change of the STarT risk group [33, 35].

Our work focused on disability being one of the core domains for patients with back pain [36], and pain intensity was chosen as a secondary outcome. This is in line with the strength of the SBT [20], but it has to be kept in mind, for example, when working with patients with pain as the major complaint, since predictive factors may differ between pain and disability [32].

The correlations determined in the presented analyses also showed similarities to results for the original version of the SBT. Stronger correlations were determined between the total score and physical measures and the sub-scores and psychological measures rather than vice versa [6].

The STarT Back approach depends on the SBT. To conduct stratified care, therapists should be upskilled through a training course in order to successfully address the complex needs of high-risk patients through the delivery of ‘psychologically informed physiotherapy’ [37]. Evidence suggests that physiotherapists trained in this manner are effective in managing around 85% of this high-risk complex patient group, but training might still be helpful to encourage therapists [38–42].

### Strengths and weaknesses

For this article, data from a control group of an RCT were analysed. In the literature, several advantages and disadvantages are described for such a proceeding [43]. An important aspect that reduced information bias was that the analysis of the STarT-G properties was intended from the design period forward: the analyses were planned by SK and KH before receiving access to the data.

The questionnaires chosen for the study were comprehensively validated, and established cut-offs were available [26, 45, 46]. On the other hand, the study sample was specifically selected. Only patients from one private health insurance were included, and, although approximately only 10% of the population are similarly insured [47], with nearly 350,000 customers, the company is large enough to represent a relevant group of people. Fewer than a fifth of the patients were lost to follow-up. This does not exceed the benchmark of 30% set for long term follow-up by the Cochrane Back and Neck Group. However, since the benchmark is set arbitrary, bias is still possible [48].

Patients with nonspecific complaints is the group which the SBT targets. In the present study, inclusion of the patients was conducted on the grounds of a search in the database of the insurance company using ICD-codes. Since a comprehensive range of diagnosis was undertaken by using codes M40 to M54, the possibility that patients with serious complaints had been included cannot be discounted. Nevertheless, inclusion of the patients for the development study of the SBT was also based on a computerized search. In the latter study, as in ours, red flag diagnoses, such as cancer, were defined as exclusion criteria [6, 44]. Despite the possibility of a heterogeneous sample being analysed, it is notable that the tool still demonstrated acceptable properties.

In various studies on the SBT, missing data were identified as a challenge when using the instrument in research. For approximately up to one-tenth of the patients in this previous work, it was not possible to determine the risk group [19, 49]. In the present study, it was possible to identify the risk group for all participants because there were no missing values, simply because the online tool used did not accept unanswered questions.

## Conclusions

Differences in the disability of patients with back pain after a period of 12 months agree with the subgroup classification determined by using the STarT-G at baseline. By adding prognostic variables to the STarT-Classification in a prognostic model, it is possible to explain nearly half of the variance observed in disability at the 12-month follow-up. Considering this information, the instrument can be used more purposefully by practitioners. Further studies to examine the predictive ability and timing of the application of the STarT-G in clinical practice and when it would be best for insurers to implement it should be conducted.

## Figures and Tables

**Fig. 3:**
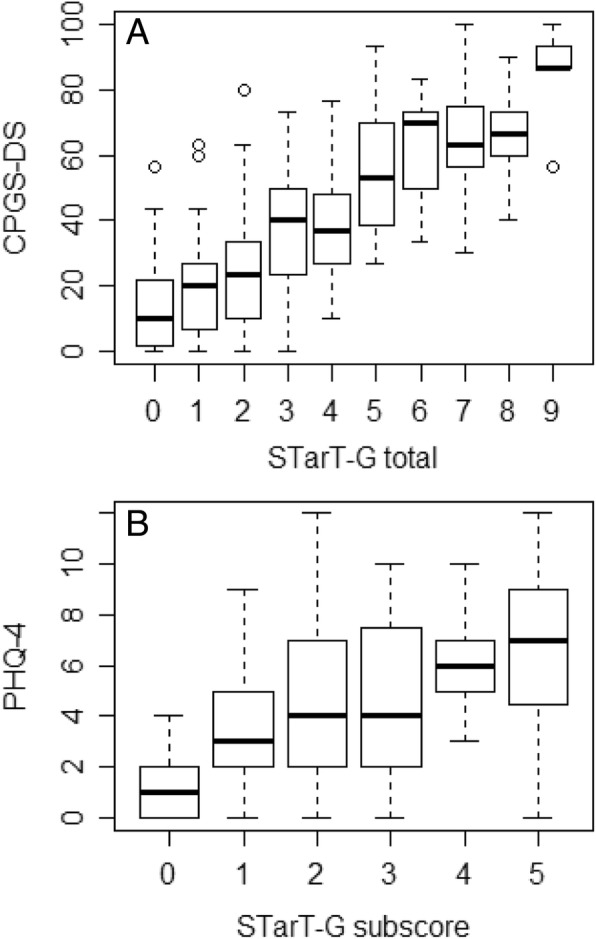
Relations between STarT-G scores and reference instruments at baseline

## Appendix 1

**Table 7:** In- and exclusion criteria checked in the insurance database, version including ICD category names

Inclusion	- Age ≥ 18 years
	- ≥ 2 cases of settlement data due to ICD codes M40-M54 (Dorsopathies)
	- Additionally one of the following three
	- ≥ 1 cases of temporary work disability in the past 12 months due to ICD codes M40-M54,
	- two opioid prescriptions or
	- settlement data due to one of the following ICD-10 codes: F32.- (Depressive episode), F33.- (Recurrent depressive disorder), F34.1-34.9 (Dysthymia, other and unspecified persistent mood [affective] disorders), F38.- (Other mood [affective] disorders), F41.2 (Mixed anxiety and depressive disorder), F43.2 (Adjustment disorders), F45.4 (Persistent somatoform pain disorder), F48.0 (Other neurotic disorders), F54 (Psychological and behavioural factors associated with disorders or diseases classified elsewhere), F62.8 (Other enduring personality changes)
Exclusion	- Care level II or III (long-term care insurance act, SGBXI)
	- ICD codes in the past 12 month (settlement data ≥ 2 for the same diagnosis or ≥ 5 different)
	- B16.-16.9 (Acute hepatitis B), B17.1 (Acute hepatitis C), B20-24 (Human immunodeficiency virus [HIV] disease), D00-D09.9 (In situ neoplasms), F00.-F09 (Organic, including symptomatic, mental disorders), F10.0-19.9 (Mental and behavioural disorders due to psychoactive substance use, not F17.0- 9), F20.-F29 (Schizophrenia, schizotypal and delusional disorders), F30.-F30.9 (Manic episode), F31.-F31.9 (Bipolar affective disorder), F42.-F42.9 (Obsessive-compulsive disorder), F60.-F60.9 (Specific personality disorders), G00-09 (Inflammatory diseases of the central nervous system), G10 (Huntington disease), G13.-G13.8 (Systemic atrophies primarily affecting central nervous system in diseases classified elsewhere), G23.-G23.9 (Other degenerative diseases of basal ganglia), G30.-32.8 (Other degenerative diseases of the nervous system), G36.-G36.9 (Other acute disseminated demyelination), G37.9 (Demyelinating disease of central nervous system, unspecified), G92 (Demyelinating disease of central nervous system, unspecified), G93.-G93.9 (Other disorders of brain), H54.0 (Visual impairment including blindness (binocular or monocular)), H91.3 (Deaf mutism, not elsewhere classified), I64 (Stroke, not specified as haemorrhage or infarction), K74.-K74.6 (Fibrosis and cirrhosis of liver), N18.0 (Chronic kidney disease), N18.3-4 (Chronic kidney disease, stage 3/stage 4), N18.9 (Chronic kidney disease, unspecified), R54 (Senility)

## Abbreviations

AUC: Area under the curve
CPGS: Chronic Pain Grade Scale
DS: Disability score
PHQ-4: Patient Health Questionnaire (4 items)
SBT: STarT Back Tool
STarT: Subgroups for Targeted Treatment
STarT-G: German version of the STarT Back Tool
